# Effect of alpha1-blockers on stentless ureteroscopic lithotripsy

**DOI:** 10.1590/S1677-5538.IBJU.2014.0478

**Published:** 2016

**Authors:** Jianguo Zhu, Yuxiang Liang, Weihong Chen, Shuxiong Xu, Yuanlin Wang, Jianxing Hu, Hui-chan He, Wei-de Zhong, Zhaolin Sun

**Affiliations:** 1 Department of Urology, The People’s Hospital of GuiZhou Provience, Guiyang, P.R. China;; 2 Department of Urology, Guangdong Key Laboratory of Clinical Molecular Medicine and Diagnostics, Guangzhou First People’s Hospital, Guangzhou Medical University, Guangzhou, China

**Keywords:** Ureteroscopy, Urinary Calculi, Lithotripsy

## Abstract

**Objective:**

To evaluate the clinical efficiency of alpha1-adrenergic antagonists on stentless ureteroscopic lithotripsy treating uncomplicated lower ureteral stones.

**Materials and Methods:**

From January 2007 to January 2013, 84 patients who have uncomplicated lower ureteral stones treated by ureteroscopic intracorporeal lithotripsy with the holmium laser were analyzed. The patients were divided into two groups, group A (44 patients received indwelled double-J stents) and group B (40 patients were treated by alpha1-adrenergic antagonists without stents). All cases of group B were treated with alpha1 blocker for 1 week.

**Results:**

The mean operative time of group A was significantly longer than group B. The incidences of hematuria, flank/abdominal pain, frequency/urgency after surgery were statistically different between both groups. The stone-free rate of each group was 100%.

**Conclusions:**

The effect of alpha1-adrenergic antagonists is more significant than indwelling stent after ureteroscopic lithotripsy in treating uncomplicated lower ureteral stones.

## INTRODUCTION

Indwelling ureteral stent has been a common urological intervention after ureteroscopic stone management in many centers, which was used to reduce upper urinary tract drainage for preventing obstruction, pain, and infection (1-3). However, stent placement is also associated with significant morbidity such as infection, encrustation, hematuria, migration and stent fracture (4, 5). With the application of small-caliber endoscopes during ureteroscopy and better intracorporeal lithotripsy devices, therefore, the necessity for placing a postoperative stent remains questionable due to the trade-off between lower incidence of postoperative ureteral stricture formation and stent-related complications.

Until now, the use of alpha1-blocker make it possible to treat lower ureteral calculi with ureteroscopic lithotripsy without indwelling ureteral stent. Alpha1-blocker is considered to be a safe and effective treatment for the small lower ureteral calculi. It was reported that alpha1-adrenergic receptors are the most abundant receptors in ureteral smooth muscle cells and it could mimic the stent-related symptoms and the lower urinary tract symptoms due to benign prostatic hyperplasia (6). To avoid the stent-associated complications, therefore, an investigation with respect to the possibility of using alpha1-blocker for the purpose of stentless ureteroscopic lithotripsy is necessary.

In view of this, we had performed a prospective study to investigate the effect of alpha1-blockers on stentless ureteroscopic lithotripsy. A series of indicators were measured to certify whether alpha1-blocker Tamsulosin could be used for the purpose of stentless ureteroscopic lithotripsy.

## MATERIALS AND METHODS

### Patients, Study Population and Ethical Statement

From January 2007 to January 2013, 84 patients who accepted ureteroscopic intracorporeal lithotripsy with the holmium laser were treated in our institution. Exclusion criteria were as follows: (1) acute renal failure, (2) chronic renal failure, (3) active urinary tract infection, (4) pregnant women, (5) history of urinary tract surgery or endoscopic treatment, (6) bladder pathology, (7) benign prostatic hyperplasia.

The patients were divided into two groups: group A included 44 patients who received indwelled double-J stents, and group B had 40 patients without stents. All cases of group B were treated with alpha1-blocker for 1 week. Institutional review board approval was obtained from the dean office and Ethics Committee of the hospital for the study, and informed consent was obtained from patients to allow their information to be used in this study in accordance with the Declaration of Helsinki.

### Study procedure

All operations were performed by the same surgeon (JG Zhu). The patients were placed in the dorsal lithotomy position under continuous epidural anesthesia. Then, ureteroscopy was inserted into bladder through urethra with direct vision. With the help of ureteral catheter, ureteroscope went up-straight along the ureter until we found the calculi. The stone was crashed by holmium laser in order to make sure the fragments were less than 0.2cm in diameter. At the end of the procedure, 44 patients of group A were submitted to indwelled double-J stents in the ureter (4.8f, 25cm), while 40 patients of group B were treated with alpha1-blocker without stents.

### Postoperative treatment

Antibiotics were routinely used to prevent from infection for 3 days. All patients were advised to take a minimum of 2L of water daily and evaluated after treatment with KUB in 2 days to identify stone clearance or not and the position of double-J stent. Patients in group B received 0.4mg tamsulosin once daily. On postoperative week 4~5, double-J stent was removed under cystoscopy under local anesthesia in group A.

### Indicators

In this study, operation time, hospital stay, and hospital charge were measured to evaluate the clinical outcomes. Furthermore, we evaluate International Prostate Symptom Score/quality of life component of IPSS (IPSS/QoL), Overactive Bladder Questionnaire (OAB-q), and Visual Analogue Pain Scale (VAPS) between the two groups at two weeks after operation. Moreover, we also evaluated complication rate in both groups at the same period (0-2 week post-operation).

### Statistical analysis

Data is presented as mean±standard deviation (SD) in the following Table. The Independent-Samples T test was used for the statistical analysis. P values less than 0.05 were considered to be statistically significant (two-tailed). Kruskal-Wallis H test was applied to assess the numeration data. All data were processed by Statistical Product and Service Solutions 13.0 (SPSS 13.0) software.

## RESULTS

### Demographic Characteristics

A total of 84 patients were enrolled in the study. Forty-four patients were assigned to A group and 40 patients to B group. In all, 84 patients completed the study as specified and 0 patients were withdrawn. By analyzing the clinical characteristics between A and B group, all clinical factors showed no significant differences between these two groups regarding age, gender, body mass index, stone diameters. Our results demonstrated that the baseline data was comparable in each group (Table-1).

**Table 1:** The demographic characteristics of patients.

Group	A (n=44)	B (n=40)	P values
Age (year)	36.59±11.26	33.05±12.72	0.737
Male/Female	26/18	22/18	0.79
Body Mass Index (Kg/m^2^)	23.66±4.06	23.93±3.81	0.771
Stone diameters (mm)	7.77±1.23	8.25±0.91	0.16
Stone-free rate	100%	100%	

### Clinical Results

Placing ureteral stents after ureteroscopy prolonged operative time, the difference of operative time between both groups was statistically significant (p=0.04) (Table-2). The mean hospital stay was 9.64±1.29 days in group A and 6.85±1.34 days in group B (p=0.0000). Stents may add to the cost of patients care due to the price of the stent and the procedure of cystoscopic stent removal (Table-2).

**Table 2:** Operation time, hospital stay and hospital charges of patients (Mean±SD).

Groups	A (n=44)	B(n=40)	p-value
Operation time (min)	41.50±6.38	26.60±6.60	p=0.04
Hospital stay(day)	9.64±1.29	6.85±1.34	p=0.04
Hospital charges (¥)	8984.91±126.00	6194.90±480.89	p=0.001

### Complication rate

Our results demonstrated that cystoscopic stent removal may raise the infection incidence. Postoperative outcomes are summarized in Table-3. The incidences of hematuria, flank/abdominal pain, frequency/urgency were statistically different between group A and group B.

**Table 3:** Comparison of postoperative complications.

Group	A (n=44)				B (n=40)					p value
	—	Light	Medium	Heavy	—	Light		Medium	Heavy	
Flank/abdominal pain	4	14	16	10	22	14		4	0	p=0.002
Hematuria	6	6	26	6	26	12		2	0	p=0.001
Urinary frequency/urgency	6	10	18	10	26	10	2		2	p=0.002

### Comparisons of IPSS/QoL, VAP scale, and OAB-q scores

By Independent-Samples T test, our results demonstrated that no significant differences on IPSS score, Qol score, OAB-q score and VAPS score were detected in both groups at pre-operation. However, we also observed that a lower score of IPSS, Qol, OAB-q e and VAPS score were show in group B than those of in group A at two weeks after operation (further details are given in Table-4).

**Table-4:** Comparisons of IPSS/QoL, VAP scale, and OAB-q scores.

Groups	A (n=44)	B(n=40)	p-value
**IPSS (storage symptom score)**			
Pre-operation	4.31±2.67	4.25±2.54	0.763
2 week Post-operation	7.79±3.42	5.88±2.74	0.025
**IPSS (voiding symptom score)**			
Pre-operation	4.70±2.61	4.65±2.57	0.814
2 week Post-operation	7.87±3.14	5.36±2.25	0.031
**Qol score**			
Pre-operation	1.92±1.67	1.83±1.50	0.541
2 week Post-operation	3.98±1.58	2.07±1.03	0.028
**OAB-q score**			
Pre-operation	7.9±1.4	8.1±1.7	0.221
2 week Post-operation	18.77±2.89	13.63±2.20	0.001
**VAPS score**			
Pre-operation	2.58±1.49	2.63±1.61	0.149
2 week Post-operation	5.06±1.42	3.02±1.70	0.041

## DISCUSSION

Ureteral stent placement after ureteroscopy is a common procedure. Without the role of stent placement, edema or mucosal inflammation might result in obstructive symptoms and become the potential for ureteral stricture. Zimskind firstly performed temporary ureteral stent placement by cystoscope three decades ago (1).From then on, the stent is considered to decrease the incidence of postoperative ureteral stricture formation (2, 3) after ureteroscopic lithotripsy. However, ureteral stents (4, 5) may also cause significant morbidity such as infection, encrustation, hematuria, migration and stent fracture. It was demonstrated that 76% of patients had urinary symptoms, 70% had severe pain and need to get analgesics, 42% had to reduce their activities and 50% of them felt less healthy in general (7). With the development of small caliber endoscopes of ureteroscopy and intracorporeal lithotripsy devices, ureteric dilation is not necessary for ureteroscopy at present. According to what we mentioned, the need for postoperative stent remains questionable.

Stentless ureteroscopy was first introduced by Hosking in 1999 (8). In the following years, however, such kind of method did not gain much popularity because of the relatively high incidence of obstructive symptoms and potential ureteral stricture after ureteroscopy. Therefore, the countermeasures for postoperative ureteral stricture formation are essential. As compared with the results obtained in patients with oral tamsulosin (0.4mg once daily), the additional benefits, in the form of hospital stay, lower hospital charges, lighter postoperative complications burden and better rational symptoms, were gained by the concurrent administration of tamsulosin. We also observed that mostly cases with longer operation durations were given stents. However, the reason that induced the longer operation durations was attributed to the procedure of stents implanting. For the patients in A group, the zebra guide wires should be inserted before the stents implanting. Therefore, the procedure of guide wire insertion resulted in longer operation durations compared with B group due to the presentation of tortuous upper ureter in some patients. In addition, a part of patients would re-implant the stents to relieve the stimuli of trigone of bladder. Therefore, the longer operation durations should be observed in patients who were given stents. Due to the same operative technique, supported treatment, clinical characteristics and care of post-operation in A and B groups, we believe that the excellent results are mainly derived from Tamsulosin. These results were partly consistent with the previous result. Recently, some prospective randomized trials have showed that post-operative pain and irritative voiding symptoms could be reduced with omission of the ureteral stent and there was no difference of stone free status between the stent and the stentless group (9-12).

Tamsulosin is a selective blocker for α1A and α1D over α1B-adrenoceptors, which are prevalent in the distal part of the ureter with a density order like α1D>α1A>α1B. It has been demonstrated that tamsulosin treatment may decrease the expulsion time and the frequency of renal colic attacks (13). Moreover, Porpiglia (14) confirmed that both nifedipine and tamsulosin could achieve an excellent expulsion rate with less additional pain medication during the treatment of distal ureteral stones. Therefore, Tamsulosin not only releases the stent-related symptoms but also improves the life quality of patients with ureteral stents (15). Alpha1-blocker can act as a selective antagonist for treating alpha1-adrenoceptor-mediated contraction of prostate, bladder, and proximal urethral smooth muscle (6). Thus, Tamsulosin is helpful for stent-related symptoms which are similar to benign prostatic hyperplasia-related symptoms. Owing to the miniaturization of endoscopes and the utilization of small-caliber holmium YAG laser, the morbidity of endourological procedures has decreased. Considering the effective role of Tamsulosin in dilating the ureteral lumen, it can be widely used in the treatment of uncomplicated lower ureteral stones combined with ureteroscopic lithotripsy.

Because the initial prospective study design was focused on effect of alpha1-blockers on stentless ureteroscopic lithotripsy, the study was presented with a number of challenges including (1 the small number of observations; 2) single center study. Therefore, a larger scale of multi-center clinical trial should be necessary in the future. In addition, some questions would arise concerning the duration for hospital stay is too long for both study groups. In China, however, the mandatory provision determined by the basic medical insurance stipulated that the preoperative examination must be done after hospitalizing, and all of these preoperative examinations should be completed for two-three days or more. It is the reason why the mean of hospital day is longer than in other countries.

## CONCLUSIONS

For patients who have uncomplicated lower ureteral stones treated by ureteroscopic intracorporeal lithotripsy with the holmium laser, the concurrent administration of tamsulosin on post- ureteroscopic intracorporeal lithotripsy should gain better clinical outcomes compared with indwelling ureteral stent. The use of alpha1-blocker make it possible to treat lower ureteral calculi with ureteroscopic lithotripsy without indwelling ureteral stent and avoid to the stent-related complications.
